# Immunization against a Conserved Surface Polysaccharide Stimulates Bovine Antibodies with Opsonic Killing Activity but Does Not Protect against *Babesia bovis* Challenge

**DOI:** 10.3390/pathogens10121598

**Published:** 2021-12-09

**Authors:** Naomi S. Taus, Colette Cywes-Bentley, Wendell C. Johnson, Gerald B. Pier, Lindsay M. Fry, Michelle R. Mousel, Massaro W. Ueti

**Affiliations:** 1 Animal Disease Research Unit, Agricultural Research Service, U.S. Department of Agriculture, Pullman, WA 99164, USA; carl.johnson@usda.gov (W.C.J.); lindsay.fry@usda.gov (L.M.F.); michelle.mousel@usda.gov (M.R.M.); massaro.ueti@usda.gov (M.W.U.); 2 Division of Infectious Diseases, Department of Medicine, Brigham and Women’s Hospital, Harvard Medical School, Boston, MA 02115, USA; ccywes@rics.bwh.harvard.edu (C.C.-B.); gpier@bwh.harvard.edu (G.B.P.); 3 Program in Vector-Borne Diseases, Department of Veterinary Microbiology and Pathology, Pullman, WA 99164, USA; 4 Paul G. Allen School for Global Animal Health, Washington State University, Pullman, WA 99164, USA

**Keywords:** *Babesia*, *Rhipicephalus* ticks, PNAG, immunization, antibody response

## Abstract

Arthropod-borne apicomplexan pathogens remain a great concern and challenge for disease control in animals and humans. In order to prevent *Babesia* infection, the discovery of antigens that elicit protective immunity is essential to establish approaches to stop disease dissemination. In this study, we determined that poly-N-acetylglucosamine (PNAG) is conserved among tick-borne pathogens including *B. bovis*, *B. bigemina*, *B. divergens*, *B. microti,* and *Babesia* WA1. Calves immunized with synthetic ß-(1→6)-linked glucosamine oligosaccharides conjugated to tetanus toxoid (5GlcNH_2_-TT) developed antibodies with in vitro opsonophagocytic activity against *Staphylococcus aureus*. Sera from immunized calves reacted to *B. bovis*. These results suggest strong immune responses against PNAG. However, 5GlcNH_2_-TT-immunized bovines challenged with *B. bovis* developed acute babesiosis with the cytoadhesion of infected erythrocytes to brain capillary vessels. While this antigen elicited antibodies that did not prevent disease, we are continuing to explore other antigens that may mitigate these vector-borne diseases for the cattle industry.

## 1. Introduction

Infection with tick-borne apicomplexan parasites such as *Babesia* spp. has a negative impact on animal and human health worldwide. The parasites are transmitted by infected ticks during their blood meal and then invade and replicate exclusively in the erythrocytes of the mammalian host (reviewed in [1,2]). Following development of the parasites to merozoites, infected erythrocytes lyse and release the merozoites, which continue the cycle by invading uninfected erythrocytes (reviewed in [1,2]). Without a protective immune response, the mammalian host suffers severe babesiosis, which is characterized by high fever, hemolytic anemia, anorexia, cachexia, hemoglobinuria, icterus, and, in some cases, death [3]. Mammalian hosts that survive acute infection become persistently infected for life and are reservoirs from which the biological vector can acquire the parasite and can subsequently transmit it to naïve hosts, as reviewed in [4].

In particular, *Babesia bovis* causes significant economic losses for livestock producers in tropical and subtropical regions because both the pathogen and its tick vector, *Rhipicephalus microplus*, are endemic. Preventive strategies for bovine babesiosis caused by *B. bovis* in these endemic regions are limited to the use of live attenuated vaccines and acaricides [1,5,6]. Drawbacks to these methods include well-recognized problems with the use of live attenuated vaccines such as possible reversion to virulence, the transmission of contaminating blood-borne pathogens, the need for a well-controlled cold chain to preserve vaccine viability, and induction of severe disease and death in cattle older than 6–9 months of age [4,5,6,7,8]. The emergence of *R. microplus* populations that are resistant to multiple acaricides as well as potential problems with environmental contamination from acaricides is also problematic [5]. Therefore, the identification of protective antigens for use in subunit vaccines is a priority for the field.

Recent work has demonstrated that a conserved polysaccharide, poly-N-acetylglucosamine (PNAG), is present on bacteria, viruses, and protozoan parasites and is hypothesized to be a potential antigen for a universal vaccine [9,10]. Natural IgG antibodies against PNAG are found in human and animal sera; however, these antibodies poorly activate the complement system and thus are not usually protective against infection [11,12,13]. However, immunization with PNAG that has been modified by reducing the acetylation of the glucosamine monosaccharides (dPNAG) or synthetic ß-(1→6)-linked glucosamine oligosaccharides conjugated to tetanus toxoid both induce antibodies that fix complement, have antimicrobial killing activity, and provide protective immunity [9,14]. Of particular interest was the finding that mice infused with polyclonal antibodies raised against a synthetic ß-(1→6)-linked glucosamine oligosaccharide conjugated with tetanus toxoid (9GlcNH_2_ –TT) and challenged with virulent *Plasmodium* parasites failed to develop cerebral disease, which is also a feature of bovine babesiosis caused by *B. bovis*; these mice also lived longer than the controls [9]. These results suggest that PNAG could be used as a target in a vaccine to prevent disease caused by *B. bovis* and possibly other *Babesia* spp. In this study, we examined several *Babesia* spp for the expression of PNAG and tested whether the immunization of calves against PNAG resulted in protection from clinical babesiosis caused by challenge with a virulent strain of *B. bovis*.

## 2. Materials and Methods

### 2.1. Immunofluorescence Assays

Direct immunofluorescence was used to demonstrate the presence of PNAG on various *Babesia* spp. Archived thin blood smear slides that were prepared as described in [15] and stored at −80 °C at the Animal Disease Research Unit, Agricultural Research Service, United States Department of Agriculture, Pullman, WA, USA, of *B. bovis* Texas [16], *B. bovis* Australia [17], *B. bigemina* Mexico [18], *B. divergens* [19,20], *B. microti* [21], and *Babesia* WA1 [21] were used for the assay. Slides were probed with IgG1 Alexa Fluor 488 conjugated fully human IgG1 monoclonal antibodies specific for PNAG (F598) or *Pseudomonas aeruginosa* alginate (F429) [11,22] at a concentration of 5.2 µg/mL for 4 h at room temperature (RT). SYTO 83 orange fluorescent nucleic acid stain (Molecular Probes) diluted to 500 nM in 0.5% bovine serum albumin in phosphate-buffered saline (BSA in PBS) with a pH of 7.4 was added to the slides without washing for the final 15 min in order to localize the parasite nuclei. The blood smears were washed, mounted with a coverslip, and antibody reactivity was visualized using confocal microscopy [9].

Indirect immunofluorescence was used to demonstrate that the sera from calves who had been immunized with 5GlcNH_2_-TT, a synthetic ß-(1→6)-linked glucosamine oligosaccharide conjugated to a tetanus toxoid, reacted with *B. bovis*. Thin smears of infected *B. bovis* red blood cells were prepared from the cultures. Slides were incubated with a 1:100 dilution of the pre- or post- immunization sera from either the control (C1587, C1588, C1598) or from the 5GlcNH_2_-TT-immunized calves (C1590, C1594, C1595) for 4 h at RT. A secondary FITC conjugated (Bethyl A10-102F) rabbit anti-bovine IgG (H+L) antibody was added to the experimental samples at a 1:100 dilution for 2 h at RT along with 500 nM of SYTO 83 that had been added without washing for the last 15 min at RT in 0.5% BSA/PBS pH 7.4. Samples were prepared as described above for confocal microscopy.

### 2.2. Pathogen and Animals

The highly transmissible Texas strain of *B. bovis* (T_2_Bo) was used in this study. This parasite strain originated from a quarantined animal in Texas [16]. Six seven-month-old Holstein steers were used in this study and were determined to be *B. bovis* free by serology and PCR targeting of the rhoptry-associated protein 1 (RAP-1) and *rap-1* gene, respectively, as previously described [23,24].

### 2.3. Immunization

Calves received three intramuscular injections using 1 1/2in needles in the left side of the neck approximately 21 days apart, at days 0, 21, and 49, with either 5GlcNH_2_-TT plus adjuvant or adjuvant alone. The immunized group received 200 µg of 5GlcNH_2_-TT (AV0328 from Alopexx Vaccine, LLC, Concord, MA, USA) in PBS plus 0.1 mL of Montanide Pet Gel A per injection (SEPPIC, Inc, Courbevoie, France). The adjuvant group received 0.1 mL Montanide Pet Gel A adjuvant diluted in PBS per injection. Montanide adjuvant was used because in a preliminary experiment, a calf who had been immunized with 5GlcNH_2_-TT and the adjuvant Specol (Thermo-Fisher Scientific, Waltham, MA, USA) failed to develop antibodies with in vitro opsonophagocytic activity (data not shown). Blood was collected prior to each immunization, and samples stored at −20 °C.

### 2.4. Enzyme-Linked Immunosorbent Assay

Total antibody titer was determined by PNAG-ELISA using sera from calves who had been immunized with either 5GlcNH_2_-TT or adjuvant alone. PNAG was isolated from *Acinetobacter baumannii*, as described in [25]. The optimization of the ELISA encompassed checkerboard titrations of PNAG and monoclonal antibody F598 to PNAG to determine the amount of PNAG that was needed to coat the ELISA plate in order to obtain maximal OD405 nm readings [11].

In brief, PNAG (0.6 µg/mL) was diluted in 0.04 M NaH_2_PO_4_ and 0.04 M Na_2_HPO_4_, which had a pH of 7.2, that had been added to the wells of Immulon 4 ELISA plates (Fisher, Hampton, NH, USA), and the samples were incubated overnight at 4 °C. The plates were washed three times with PBS containing 0.05% Tween 20 (wash buffer). The antigen-coated plates were blocked with PBS plus 2.5% bovine serum albumin for 1 h at 37 °C. PBS containing 1% BSA and 0.05% Tween 20 (dilution buffer) was used to dilute all of the sera. To determine antibody titer, pre- and post-immunization sera from immunized or adjuvant groups were diluted 1:100 with subsequent two-fold dilutions. Diluted sera were added to the wells and were incubated for 1 h at 37 °C. Plates were washed three times with wash buffer, and a 1:5000 dilution of alkaline phosphatase-conjugated rabbit anti-bovine IgG in dilution buffer was added and incubated for 1 h at RT. Plates were washed as before, a 4-nitrophenyl phosphate disodium salt hexahydrate tablet (Sigma) was dissolved in 15 mL of 20 mM NaHCO_3_, 28 mM Na_2_CO_3_, and 1 mM MgCl_2_ (substrate buffer), and this was then added to the wells. The plates were incubated in the dark for 1 h at 37 °C, and the optical density (OD) at 405 nm was measured. End-point titer was calculated by subtracting the average negative OD from the average sample OD, with a value above 0.4999, considered positive and was reported as the reciprocal of the highest dilution providing a positive value.

### 2.5. Opsonophagocytic Killing Assay. In Vitro Killing of Staphylococcus aureus

MN8 was performed as previously described [9,26]. Controls included mAb F429 against alginate with human phagocytic cells and 10% bovine complement, mAb F598 against PNAG and 10% bovine complement without phagocytic cells, mAb F598 and phagocytic cells without bovine complement, and mAb F598 with phagocytic cells and complement. Percent killing was calculated as the ratio of the number of colony-forming units (CFU) in the tubes containing bacteria as well as the complement, phagocytic cell, and post-immunization sera to the number of CFU in the tubes containing bacteria, complement, phagocytic cell, and pre-immunization sera.

### 2.6. Challenge with B. bovis Texas T2Bo Stabilate

Twenty-seven days after the last immunization, the calves were challenged via the intravenous injection of 10^7^ *B. bovis*-infected erythrocytes in 5 mL of diluent (Puck’s Saline G with 10% normal bovine serum). Rectal temperature, appetite, attitude, packed cell volume (PCV), and parasitemia, were monitored daily via PCR beginning on day 5 post-infection. When a rectal body temperature > 40.3 °C [27] was measured, the calves were administered flunixin meglumine (1.1–2.2 mg/kg; Merck Animal Health, Kenilworth, NJ, USA). When the animals were off-feed or lethargic, oral electrolytes were offered twice a day in order to avoid/combat dehydration (due to pyrexia and decreased water intake) and to provide some energy and electrolyte intake (due to anorexia) as well as to help determine the overall status of the animals. Reluctance or refusal to take the electrolytes indicated that animals were experiencing the worsening of acute disease. The calves were euthanized if the body temperature remained >40.3 °C for 3 days and/or if the PCV dropped below 14% and/or if inappetence and lethargy were progressing.

### 2.7. PCR to Detect B. bovis Post-Challenge

Nested PCR was conducted as previously described in order to detect rap-1 [23,24]. Quantitative PCR was used to detect the merozoite surface antigen 1 gene (msa-1) and was conducted essentially as described; however, the final reaction volume was 25 µL [28,29].

### 2.8. Statistical Analysis

PNAG antibody titers were analyzed using a mixed model with the fixed effects of treatment and the day of sampling, and animal was used as the random variable. The parameters for infection in the vaccinated and control groups of calves were compared with Student’s *t*-test (peak decrease in packed cell volume, days to develop fever, peak parasite copy numbers) and the Mann–Whitney rank sum test (days to detection of parasitemia).

### 2.9. Ethics Statement

This study was approved by the Institutional Animal Care and Use Protocol Committees of the University of Idaho, Moscow, Idaho (protocol #2018-16), in accordance with institutional guidelines based on the U.S. National Institutes of Health (NIH) Guide for the Care and Use of Laboratory Animals.

## 3. Results

### 3.1. Expression of PNAG on Babesia parasites

**Figure 1:**
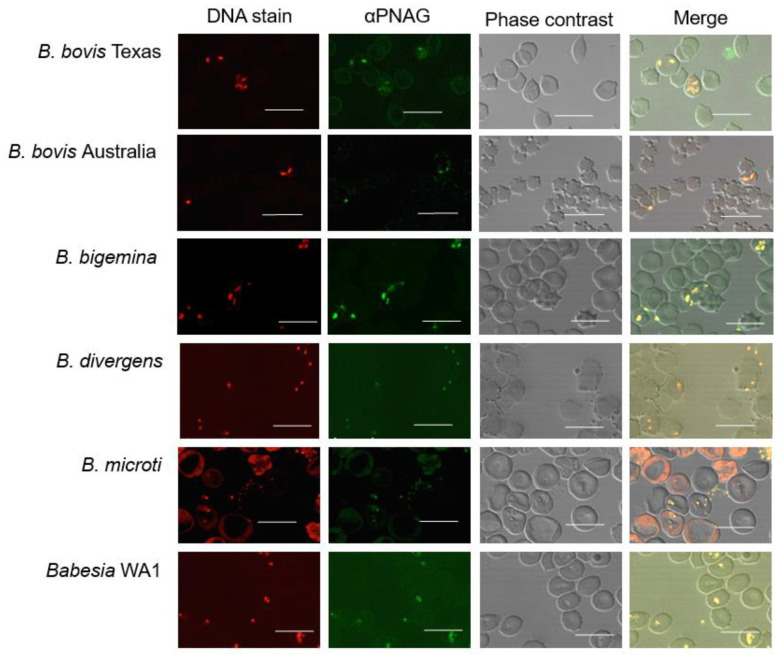
Conservation of poly-N-acetyl-glucosamine (PNAG) in *Babesia* spp. demonstrated using direct immunofluorescence on infected blood smears. DNA stain = *Babesia* DNA detected using SYTO 83; αPNAG = Alexafluor 488-conjugated anti-PNAG monoclonal antibody; phase contrast = light micrograph; merge = merged image of panels DNA stain and αPNAG columns. White bars = 10 µm.

Immunofluorescence assays using the PNAG-specific mAb F598 demonstrated that PNAG was present on the blood stages of the bovine *Babesia* spp., *B. bovis,* and *B. bigemina*; the zoonotic pathogen, *B. divergens*; and the human parasites *B. microti* and *Babesia* WA1 (Figure 1, αPNAG, green). In contrast, isotype control mAb F429 showed no reactivity with the *Babesia parasites* (Supplemental Figure S1). The co-localization of the anti-PNAG antibodies with *Babesia* was determined by DNA labeling using SYTO 83 (Figure 1, DNA stain, red). Merged images showed the specific reactivity of mAb F598 to the *Babesia parasites* (Figure 1, Merge, yellow).

### 3.2. Characterization of PNAG-Specific Antibodies in Immunized Calves

**Figure 2:**
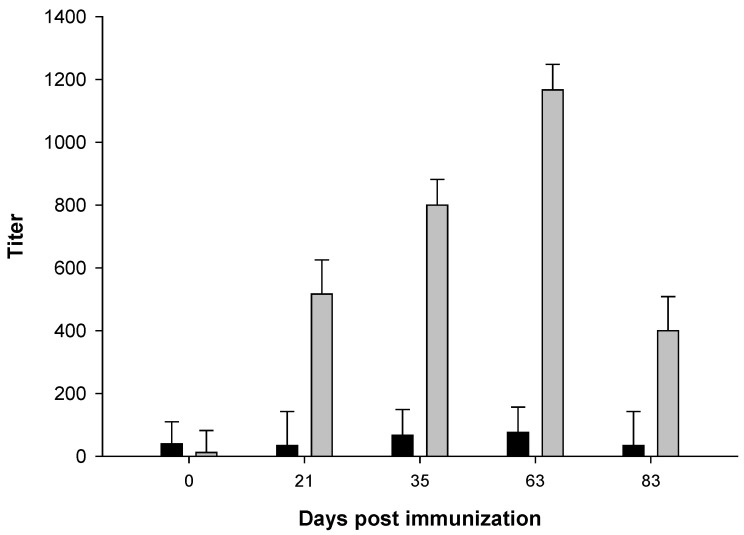
Stimulation of PNAG-specific antibodies following immunization with 5GlcNH_2_-TT plus adjuvant (light bars) vs. adjuvant only group (dark bars). *p* < 0.001, ANOVA.

**Figure 3:**
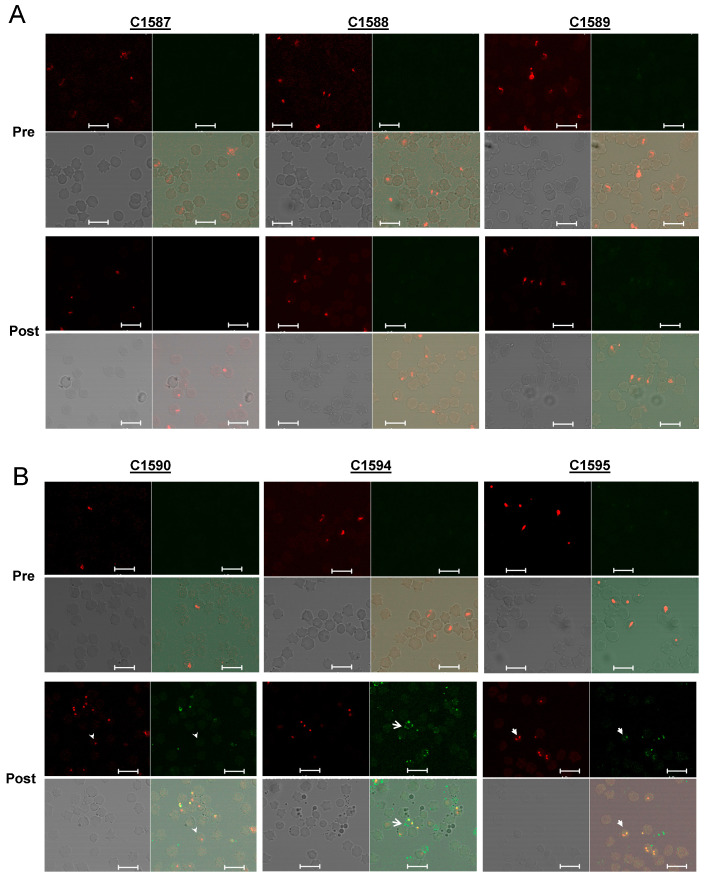
Recognition of *B. bovis* blood stages by sera from immunized cattle demonstrated using indirect immunofluorescence. Each set of four panels arranged starting at upper left going clockwise are SYTO 83 DNA stain, calf serum, merged image, phase contrast. (**A**) Reactivity of sera from control calves with thin blood smears of *B. bovis*-infected red blood cells. (**B**) Reactivity of sera from 5GlcNH_2_-TT-immunized calves with thin blood smears of *B. bovis*-infected red blood cells. Arrow heads in C1590 Post indicate parasite without serum reactivity. Arrows in C1594 Post indicate serum reactivity without DNA staining. Closed arrows in C1595 indicate co-localization of DNA stain and serum reactivity. Pre = Pre-immune sera; Post = sera 72d post-immunization. White bars are 10 µm.

**Figure 4:**
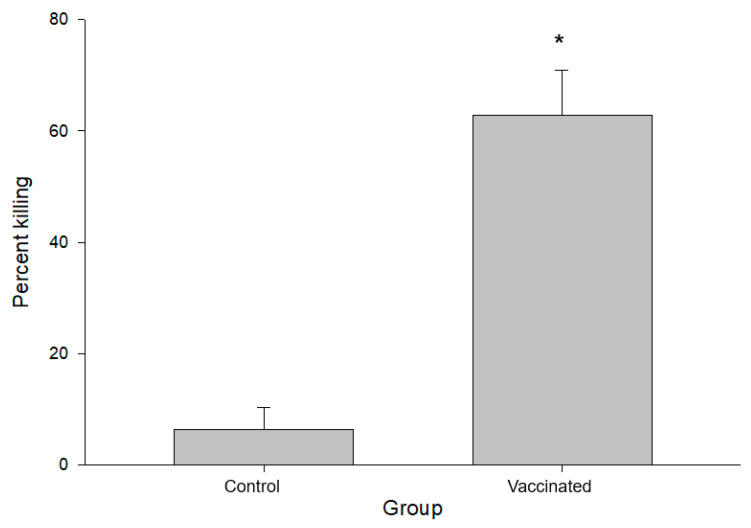
Opsonophagocytic killing of *Staphylococcus* *aureus* MN8 by sera 35d post vaccination. * *p* < 0.001, Student’s *t*-test.

All three animals who had been immunized with 5GlcNH_2_-TT developed specific antibodies against PNAG, which were measured using ELISA, while the control animals did not (*p* < 0.001, Figure 2). Immunofluoresence assays using pre-immune sera (Figure 3A,B, Pre) and sera from cattle in the adjuvant group (Figure 3A, Post) showed no antibody reactivity against PNAG on the *B. bovis* Texas strain. In contrast, sera from the immunized group recognized PNAG on the *B. bovis* Texas strain (Figure 3B, Post). Immunization also induced antibodies with opsonophagocytic killing activity against *S. aureus* (Figure 4). 

### 3.3. Development of Babesiosis in Immunized Calves

**Figure 5:**
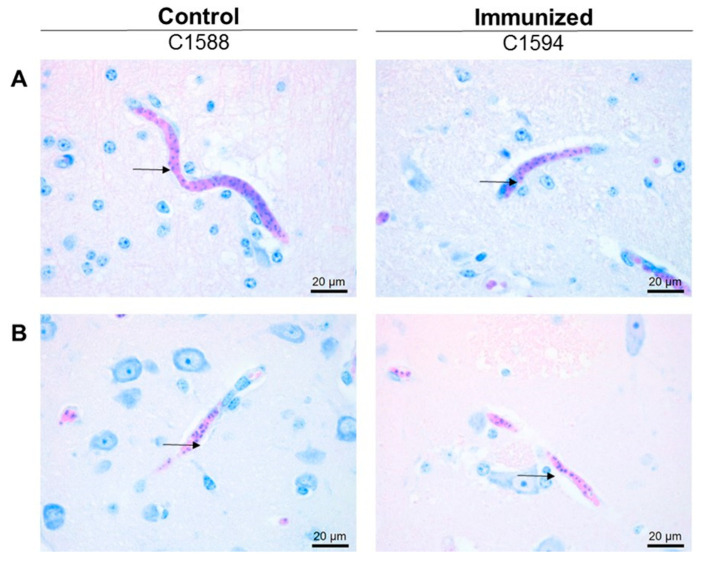
Detection of *B. bovis*-infected erythrocytes in the capillaries of the (**A**) cerebrum and (**B**) midbrain of an adjuvant-only control calf (Control) and a PNAG-immunized calf (Immunized) challenged with *B. bovis*. Arrows indicate *B. bovis*-infected erythrocytes. Images are representative of all calves.

All of the animals in both the vaccinated and control groups suffered severe clinical signs of bovine babesiosis regardless of the antibody titer or killing ability of the antibodies (Table 1 and Tables S1–S3). Severe disease caused by *B. bovis* is the result of cerebral babesiosis, in which parasitized erythrocytes are sequestered in the capillary vessels of the brain. Histological evaluation of the brain from all of the calves revealed high levels of infected *B. bovis* erythrocytes in the capillaries of the cerebrum and midbrain, indicating that the antibody to PNAG did not affect the sequestration of the *B. bovis*-infected erythrocytes. Representative histological images from a control calf (Figure 5 Control) and vaccinated calf are shown (Figure 5 Immunized).

## 4. Discussion

The immune control of *B. bovis* infection in cattle is thought to include both innate and adaptive responses [4,30]. In young calves who are up to about 6–10 months of age, resistance to severe acute disease is mediated by splenic innate immune cells that also lead to adaptive responses, CD4+ T-cells, and memory B-cells that protect against disease from subsequent infections as the animal ages. This innate immune resistance allows the safe use of live vaccines in young calves. Immunologically naïve older cattle generally do not display a strong innate immune response and succumb to acute disease [4,8,30,31,32], which precludes the use of live vaccines in older animals.

Subunit vaccines avoid the problem of live vaccine-induced disease, and studies have looked at using recombinant proteins alone, as reviewed in [30], or recombinant proteins followed by a boost with a virus vector encoding the antigen [33] in order to stimulate protection against the clinical disease that is caused by *B. bovis*. Despite the stimulation of antigen-specific CD4+ T-cells and antibodies, none of the vaccine regimens resulted in protection from disease when the cattle were challenged with virulent *B. bovis* [30,33], illustrating the complexity of developing vaccines against *B. bovis* [6]. 

Early work, most of which was focused of the role of humoral immunity against babesiosis, demonstrated that passively transferred antibodies protected the cattle against disease caused by homologous strains of *B. bovis* [4]. We sought to identify an antigen conserved among different strains and species of *Babesia* that could be used to provide a broadly protective antibody response against severe clinical disease.

Numerous studies have demonstrated the induction of protective antibody responses against bacteria by immunization with dPNAG or synthetic ß-(1→6)-linked glucosamine oligosaccharides that have been conjugated to carrier proteins (reviewed in [10]) as well as a few eukaryotic pathogens, including the apicomplexan parasite *Plasmodium berghei* [9]. Specifically, the anti-PNAG antibodies prevented cerebral malaria, decreased parasitemia, and significantly prolonged the survival of mice who had been infected with *P. berghei* [9]. Because *B. bovis*-infected red blood cell sequestration in brain capillaries is a prominent feature of clinical babesiosis in infected cattle, we hypothesized that antibodies to PNAG would provide protection against *B. bovis,* similar to what was seen in mice.

The immunization of calves with PNAG stimulated high-titer, specific antibody responses with opsonophagocytic killing activity against a control bacterium, *S. aureus* MN8, which is known to express PNAG. However, despite the documented display of PNAG by the parasite, this antibody response did not protect calves who had been challenged with *B. bovis*-infected erythrocytes against the severe clinical manifestation of bovine babesiosis. All of the PNAG-immunized animals developed clinical babesiosis with parasite sequestration in brain capillaries and peripheral parasite loads that were indistinguishable from the control animals. This result differs from the results of the study by Cywes-Bentley et al. [9]. 

The protection mechanism that is induced by PNAG antibodies is achieved via the deposition of complement components onto the target cell(s), leading to the opsonic killing of the pathogen [9,10,14]. Thus, this requires babesial PNAG to be physically accessible to the antibodies. Although the blood stages of *Babesia* exit the erythrocytes in order to infect new red blood cells in order to perpetuate the merozoite propagation cycle, it is possible that the antigen is not surface exposed, either due to location or because it is masked by known surface proteins such as merozoite surface antigens.

Finally, the immune correlates of disease protection, including the full elucidation of the role of different T-cell subsets in the protection against bovine babesiosis, remain elusive. Interestingly, Jaramillo Ortiz et al. observed antigen-specific CD8+ T-cells in cattle who had been immunized with a live vaccine [33]. However, since *B. bovis* only resides in erythrocytes, it is unclear what role the CD8+ T-cells have in controlling disease. More studies examining the mechanisms of innate and acquired immune responses in calves to *B. bovis* infection are needed and will lead to improved vaccination strategies in the efforts to control babesiosis in cattle and to prevent economic losses for livestock producers.

## 5. Conclusions

Developing safe and effective vaccines to protect cattle from babesiosis is a top priority for animal health researchers. Identifying conserved antigens that can induce immunity against different *Babesia* species and strains is challenging. Poly-N-acetyl-glucosamine is a conserved surface polysaccharide that is expressed by a great diversity of organisms, including the apicomplexan parasites *P. berghei* and *P. falciparum,* and antibodies against PNAG provided protection from disease in a mouse malaria model. In our study, we showed that *Babesia* spp express PNAG, making it an attractive and potential target for a universal vaccine against babesiosis. Despite the development of anti-PNAG antibodies in calves following immunization with 5GlcNH_2_-TT, no protection against acute babesiosis was detected. However, it was important to experimentally test the possibility that anti-PNAG antibodies could provide some protection from disease, and the result of this work does not preclude testing as to whether PNAG is useful against other protozoan parasites.

## Figures and Tables

**Table 1:** Development of babesiosis in calves inoculated with *B. bovis*.

	Adjuvant ^1^	PNAG ^2^	Test, *p* Value
Parameter			
Median days to detect parasitemia	6 DPI ^3^	7 DPI	Mann–Whitney rank sum test, *p* = 0.2
Mean peak decrease in PCV ^4^	37.76%	45.43%	Student’s *t*-test, *p* = 0.537
Mean days to develop fever ≥ 39.4 °C	7.7	8.3	Student’s *t*-test, *p* = 0.519
Mean peak parasite copy numbers ± SD	1.82 × 10^6^ ± 2.26 × 10^6^	8.64 × 10^5^ ± 2.26 × 10^5^	Student’s *t*-test, *p* = 0.508

^1^ Adjuvant = animals immunized with adjuvant only, ^2^ PNAG = animals immunized with 5GlcNH_2_-TT. ^3^ DPI = days post inoculation with *B. bovis*, ^4^ PCV = packed red cell volume, SD = standard deviation.

## Data Availability

Not applicable.

## Supplementary Materials

The following are available online at https://www.mdpi.com/article/10.3390/pathogens10121598/s1, Figure S1: Blood smears probed with isotype control (F429) and Babesia DNA labeled with SYTO 83 (red fluorescence), Table S1: Development of babesiosis in individual calves inoculated with B. bovis, Table S2: Clinical parameters measured in calves following intravenous inoculation of T2Bo B. bovis infected erythrocyte. Table S3: Detection of T2Bo B. bovis DNA in peripheral blood following inoculation of infected erythrocytes.

## Abbreviations

PNAG	poly-N-acetylglucosamine
dPNAG	deactylated poly-N-acetylglucosamine
TT	tetanus toxoid
BSA	bovine serum albumin
PCV	packed cell volume
