# Supplementary material for: Immunization against a Conserved Surface Polysaccharide Stimulates Bovine Antibodies with Opsonic Killing Activity but Does Not Protect against Babesia bovis Challenge
Source: Pathogens. 2021 Dec 9;10(12):1598. doi: 10.3390/pathogens10121598 (PMC8709247; doi:10.3390/pathogens10121598)
Supplement: Supplementary file 1 [file pathogens-10-01598-s001.zip › pathogens-1472949-supplementary/Figure S1.pptx]

## Slide 1
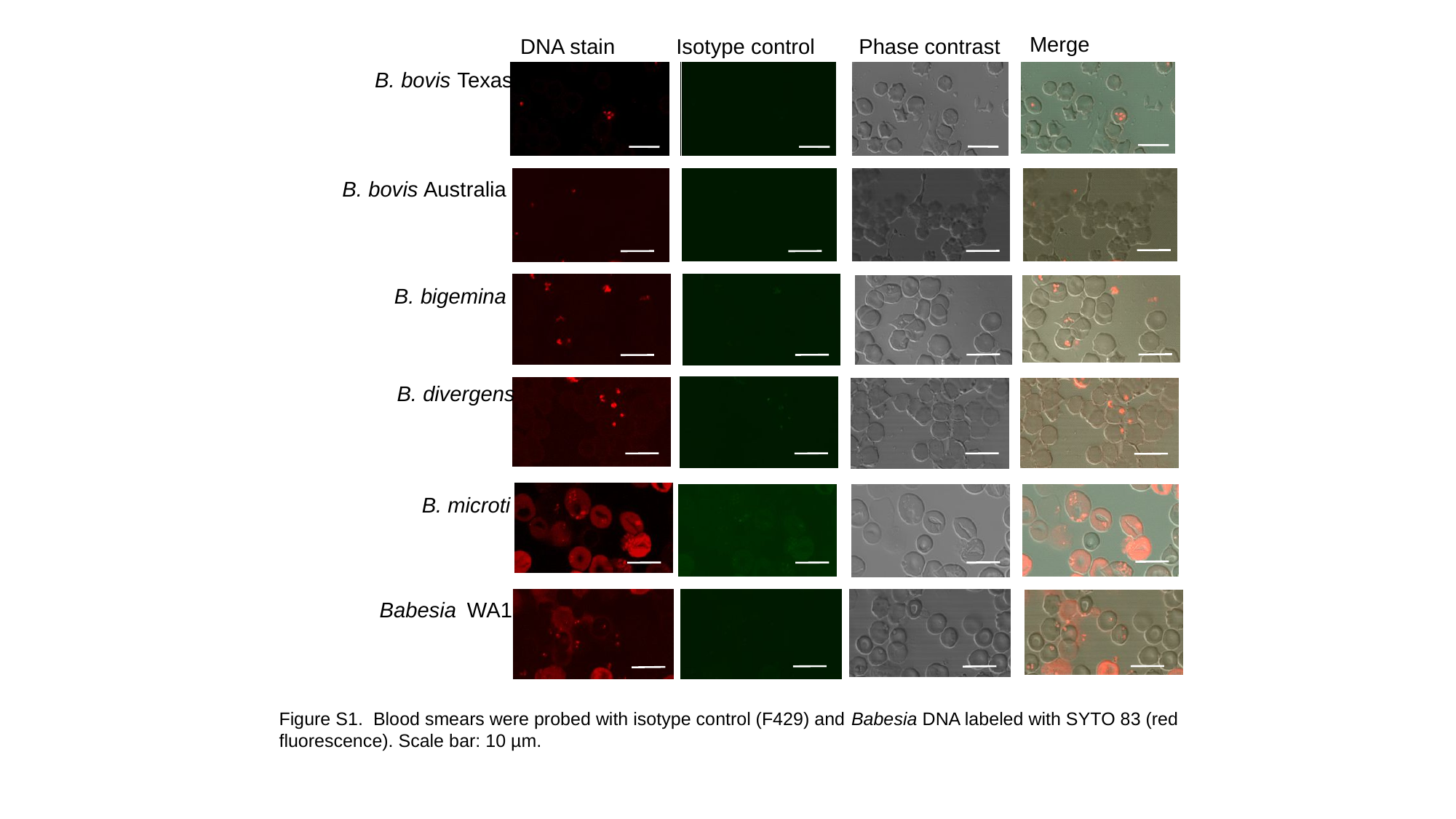

Merge
DNA stain
Isotype control
Phase
contrast
B.
b
ovis
Texas
B.
b
ovis
Australia
B.
b
igemina
B.
d
ivergens
B.
microti
Babesia
WA1
Figure S1. Blood smears were probed with isotype control (F429) and Babesia DNA labeled with SYTO 83 (red fluorescence). Scale bar: 10 µm.
