# Supplementary material for: Immunization against a Conserved Surface Polysaccharide Stimulates Bovine Antibodies with Opsonic Killing Activity but Does Not Protect against Babesia bovis Challenge
Source: Pathogens. 2021 Dec 9;10(12):1598. doi: 10.3390/pathogens10121598 (PMC8709247; doi:10.3390/pathogens10121598)
Supplement: Supplementary file 1 [file pathogens-10-01598-s001.zip › pathogens-1472949-supplementary/Table S1.pdf]

Table S1 Development of babesiosis in individual calves inoculated with *B. bovis*

		First detection of parasitemia[2] (DPI)	Peak parasitemia copies/ml[3] (±SD)	Decrease in PCV (%)	DPI to temp ≥ 39.4 °C
Animal ID	Group[1]				
1587	Adjuvant	6	7.15×10^5 (1.99×10^5)	53.3	8
1588	Adjuvant	6	4.42×10^6 (1.74×10^6)	16.7	9
1589	Adjuvant	7	3.18×10^5 (1.26×10^5)	43.3	6
1590	PNAG	7	9.78×10^5 (2.92×10^5)	43.3	9
1594	PNAG	7	6.04×10^5 (1.73×10^5)	51.6	8
1595	PNAG	8	1.01×10^6 (4.08×10^5)	41.4	8

[1]Adjuvant = animals immunized with adjuvant only, PNAG = animals immunized with 5GlcNH2-TT.

[2]Parasite DNA detected using nested PCR, DPI = Days post inoculation with *B. bovis*,

[3] Real time quantitative PCR, SD = Standard deviation

PCV= Packed red cell volume.
