# Supplementary material for: Immunization against a Conserved Surface Polysaccharide Stimulates Bovine Antibodies with Opsonic Killing Activity but Does Not Protect against Babesia bovis Challenge
Source: Pathogens. 2021 Dec 9;10(12):1598. doi: 10.3390/pathogens10121598 (PMC8709247; doi:10.3390/pathogens10121598)
Supplement: Supplementary file 1 [file pathogens-10-01598-s001.zip › pathogens-1472949-supplementary/Table S2.pdf]

Table S2 Clinical parameters measured in calves following intravenous inoculation of T_2Bo *B. bovis* infected erythrocytes

	1587 [1]		1588 [1]		1589 [1]		1590 [2]		1594 [2]		1595 [2]	
	PCV (%)	Temp (°C)	PCV (%)	Temp (°C)	PCV (%)	Temp (°C)	PCV (%)	Temp (°C)	PCV (%)	Temp (°C)	PCV (%)	Temp (°C)
DPI												
0	30	ND	30	ND	28	ND	30	ND	31	ND	29	ND
1	29	38.2	30	38.2	29	38.6	29	38.8	30	38.8	29	38.7
2	ND	38.2	ND	38.3	ND	38.3	ND	38.6	ND	38.8	ND	38.3
3	30	38.3	32	38.3	30	38.2	31	37.6	31	38.4	31	38.3
4	29	38.3	32	38.4	31	38.3	31.5	38.0	30	38.6	29	38.4
5	29	38.7	31	38.7	30.5	38.3	29.5	38.0	30.5	38.9	29	37.6
6	30	38.7	32	38.3	31	39.7	31	38.7	31	39.3	31	38.7
7	29	38.6	30	38.4	30.5	39.3	29	38.7	30	39.2	31	39.3
8	25	40.4	32	39.2	29	39.8	27	39.2	27	39.7	32	39.7
9	22	40.9	29	40.3	28	39.9	25	39.9	26	40.6	26	40.4
	22	40.2	27	40.1	24	40.6	25	40.1	24	40.8	24	40.3
10	24	40.4	25	40.9	24	40.6	24	40.4	24	40.2	23	40.7
	ND	40.6	ND	41.3	ND	39.6	ND	40.3	ND	40.2	ND	40.2
	21	40.6	24	41.4	ND	ND	ND	ND	ND	ND	ND	ND
11	19	40.7	25	41.4	20	40.9	20	40.5	20	40.6	20	41.0
	ND	40.5	ND	40.7	ND	40.6	ND	40.3	ND	40.2	ND	40.4
	ND	41.2	ND	41.8	ND	41.2	ND	41.0	ND	41.2	ND	41.0
12	14	40.0	25	40.3	15	40.2	17	39.6	15	39.7	17	40.2

[1] Calves immunized with adjuant, [2] Calves immunized with 5GlcNH2-TT, PCV= packed cell volume, DPI= days post intravenous inoculation, ND= not determined.
