# Supplementary material for: Immunization against a Conserved Surface Polysaccharide Stimulates Bovine Antibodies with Opsonic Killing Activity but Does Not Protect against Babesia bovis Challenge
Source: Pathogens. 2021 Dec 9;10(12):1598. doi: 10.3390/pathogens10121598 (PMC8709247; doi:10.3390/pathogens10121598)
Supplement: Supplementary file 1 [file pathogens-10-01598-s001.zip › pathogens-1472949-supplementary/Table S3.pdf]

Table S3 Detection of T_2Bo *B. bovis* DNA in peripheral blood following inoculation of infected erythrocytes.

A. Nested PCR for *B. bovis rap1*

DPI	1587 [1]	1588 [1]	1589 [1]	1590 [2]	1594 [2]	1595 [2]
0	ND	ND	ND	ND	ND	ND
1	ND	ND	ND	ND	ND	ND
2	ND	ND	ND	ND	ND	ND
3	ND	ND	ND	ND	ND	ND
4	ND	ND	ND	ND	ND	ND
5	ND	ND	ND	ND	ND	ND
6	Pos	Pos	Neg	Neg	Neg	Neg
7	Pos	Pos	Pos	Pos	Pos	Neg
8 *	Pos	Pos	Pos	Pos	Pos	Pos

[1] Calves immunized with adjuvant only, [2] Calves immunized with 5GlcNH2-TT, DPI= days post intravenous inoculation, ND= not determined

B. qPCR for *B. bovis msa1* (copies/ml blood)

	1587 [1]		1588 [1]		1589 [1]		1590 [2]		1594 [2]		1595 [2]	
	Mean	SD	Mean	SD	Mean	SD	Mean	SD	Mean	SD	Mean	SD
DPI												
0	ND		ND		ND		ND		ND		ND	
1	ND		ND		ND		ND		ND		ND	
3	ND		ND		ND		ND		ND		ND	
4	ND		ND		ND		ND		ND		ND	
5	ND		ND		ND		ND		ND		ND	
6	ND		ND		ND		ND		ND		ND	
7	ND		ND		1.35E+01	6.21E+00	ND		7.07E+00	4.15E+00	ND	
8	6.74E+00	4.769943	6.86E+01	2.23E+01	1.28E+02	1.28E+02	4.45E+01	8.22E+00	8.53E+01	3.14E+00	1.14E+01	1.12E+00
9	8.09E+04	1.41E+04	1.52E+05	8.93E+04	1.62E+05	7.82E+04	1.06E+05	4.66E+04	1.89E+05	5.10E+04	3.52E+05	3.41E+05
10	7.15E+05	1.99E+05	5.14E+05	2.43E+05	2.39E+05	2.75E+04	9.78E+05	2.92E+05	3.23E+05	2.73E+04	3.42E+05	6.15E+03
11	3.58E+05	2.10E+04	4.42E+06	1.74E+06	1.79E+05	1.59E+04	7.97E+05	2.95E+05	6.04E+05	1.73E+05	1.01E+06	4.08E+05
12	5.98E+05	1.54E+05	9.31E+05	2.49E+05	3.18E+05	1.26E+05	6.49E+05	4.81E+05	3.12E+05	3.57E+04	7.97E+05	2.95E+05

[1] Calves immunized with adjuvant only, [2] Calves immunized with 5GlcNH2-TT, DPI= days post intravenous inoculation, ND= not determined
